# Successful twin pregnancy in a 38-year-old woman with Pompe disease despite interruption of enzyme replacement therapy (ERT)

**DOI:** 10.1186/1471-2474-14-S2-P8

**Published:** 2013-05-29

**Authors:** N Tiling, L Bosanska, G Gossing, H Krieg, A Krebs, S Rosseau, H Schütte, K Irlbacher, U Plöckinger

**Affiliations:** 1 Charité Universitätsmedizin Berlin, Berlin, Germany

## Introduction

Clinical features of Pompe disease are limb-girdle muscle weakness and respiratory insufficiency. Data on pregnancy with or without ERT are rare. Herein, we report on a twin pregnancy in a 38-year-old primiparous who declined ERT during pregnancy.

## Results

After successful insemination of 2 foetuses, ERT was stopped at the patient’s request. Investigations were performed before and during pregnancy until week 30. The Walton/Gardner Medwin Scale was stable during the preceding 2y on ERT and during pregnancy. Echocardiography demonstrated normal left-ventricular function. No changes were observed in creatine kinase (CK) levels, GAA activity, or quality of life (SF-36). In contrast, pulmonary and functional scores clearly deteriorated: FEV1 (58% → 54%) and supine VC (61 → 55%); 6-min walk test 324 → 240m (73%); climbing 4 steps 4 → 5.1sec (128%); standing from supine 4.5 → 19sec (433%; due to back pain); 10m walking 8 → 10m (125%). Growth-curves for the dichorionicdiamniote twins were normal. At week 34, HELLP-syndrome (hemolysis, elevated liver function test, low platelet counts) was diagnosed due to increased ALT 618U/l (N<35), reduced thrombocyte count [95/nl (N>150)] and peripheral edema. Subsequently, a cesarean section was performed under combined spinal epidural anesthesia. No intra or postoperative pulmonary complications occurred. Two mature, healthy boys, with normal GAA were delivered. Peripartal evaluation of the patient’s GAA showed a uniquely normal level.

## Discussion

We describe the first in vitro fertilisation and twin pregnancy in a patient with Pompe disease with interruption of ERT during pregnancy. The observed clinical deterioration may have resulted from a combination of treatment interruption and pregnancy-related restrictions on pulmonary/motor function. The patient’s back pain may be related to an exacerbated strain on weak spinal muscles due to the twin pregnancy. No transfer of GAA from the foetus to the mother was observed during pregnancy, but peripartal transfer occurred. HELLP-Syndrome is thought to be unrelated to Pompe since it is a common complication in elderly primiparous.

## Conclusion

For a patient that has a stable and reasonably good status before pregnancy, the interruption of ERT may be possible without deleterious effects. However, close follow-up is required.

